# Identification and internal validation of models for predicting survival and ICU admission following a traumatic injury

**DOI:** 10.1186/s13049-018-0563-5

**Published:** 2018-11-12

**Authors:** Rebecca J. Mitchell, Hsuen P. Ting, Tim Driscoll, Jeffrey Braithwaite

**Affiliations:** 1 Australian Institute of Health Innovation, Macquarie University, Level 6, 75 Talavera Road, Sydney, NSW 2109 Australia; 2 Sydney Medical School, The University of Sydney, Sydney, Australia

**Keywords:** Trauma, Trauma severity, 90-day mortality, International classification of diseases

## Abstract

**Background:**

Measures to improve the accuracy of determining survival and intensive care unit (ICU) admission using the International Classification of Injury Severity Score (ICISS) are not often conducted on a population-wide basis. The aim is to determine if the predictive ability of survival and ICU admission using ICISS can be improved depending on the method used to derive ICISS and incremental inclusion of covariates.

**Method:**

A retrospective analysis of linked injury hospitalisation and mortality data during 1 January 2010 to 30 June 2014 in New South Wales, Australia was conducted. Both multiplicative-injury and single-worst-injury ICISS were calculated. Logistic regression examined 90-day mortality and ICU admission with a range of predictor variables. The models were assessed in terms of their ability to discriminate survivors and non-survivors, model fit, and variation explained.

**Results:**

There were 735,961 index injury admissions, 13,744 (1.9%) deaths within 90-days and 23,054 (3.1%) ICU admissions. The best predictive model for 90-day mortality was single-worst-injury ICISS including age group, gender, all comorbidities, trauma centre type, injury mechanism, and nature of injury as covariates. The multiplicative-injury ICISS with age group, gender, all comorbidities, injury mechanism, and nature of injury was the best predictive model for ICU admission.

**Conclusions:**

The inclusion of comorbid conditions, injury mechanism and nature of injury, improved discrimination for both 90-day mortality and ICU admission. Moves to routinely use ICD-based injury severity measures, such as ICISS, should be considered for hospitalisation data replacing more resource-intensive injury severity classification measures.

**Electronic supplementary material:**

The online version of this article (10.1186/s13049-018-0563-5) contains supplementary material, which is available to authorized users.

## Background

The International Classification of Injury Severity Score (ICISS) is one of a number of indices that can be used to estimate injury severity [1–4]. The ICISS has previously been shown to provide good estimates of survival following injury [1, 5]. It also has a practical advantage [6] as it is relatively easily derived by calculating Survival Risk Ratios (SRRs) for injury diagnosis classifications using hospital administrative data and an indicator of mortality. Both in-hospital [5, 7] and post-discharge mortality up to 90-days post-admission [8] have been used to indicate survival. The ICISS has largely been derived using data from trauma centre registries [2, 3] which generally record information on more severely injured individuals, and have higher mortality rates (around 5%) compared to all injury admissions (around 1–2%) [7, 8]. In addition, the ICISS has been estimated using pooled diagnosis-specific survival probabilities from hospitalisation data across several high-income countries using in-hospital mortality to indicate survival [9].

Assessments of the predictive ability of ICISS values to determine survival have found that the SRR of the worst-injury is often a better predictor of survival than a multiplicative combination of SRRs for all injury diagnoses [10, 11]. Previous studies have also examined the inclusion of other factors that may impact on survival, such as age, gender, pre-injury comorbid conditions, and mechanism of injury. None have considered whether including type of trauma service improves predictive ability [1–3, 7, 8, 10]. Previous research has indicated that treatment at a major trauma service can provide a survival advantage [12, 13], but it is unclear whether the accuracy of ICISS to indicate post-discharge survival could be improved by including type of trauma service as a covariate.

Various factors are associated with an injured individual being admitted to an Intensive Care Unit (ICU), including age, gender, comorbid health conditions, and injury mechanism [14–16]. However, there has been limited examination of ICISS as a tool to assist in predicting ICU admission [5]. Predicting which patients may require admission to ICU based on their injury severity will assist in determining resource use and is an additional health outcome indicator. Assessment of whether multiplicative-injury or worst-injury ICISS is a better predictor of ICU admission for trauma patients on a population-wide basis is needed.

Different approaches have previously been used to internally validate SRRs estimated from in-hospital records. These have largely involved using the split-sample approach, where the data are randomly spilt into two (or more) parts, with one dataset acting as the training dataset where the model is developed, and the other dataset(s) acting as the testing dataset(s) where predictive accuracy is assessed [6, 10], or using a bootstrapping approach where the model is developed on the full dataset and bootstrapping is applied to assess performance [7, 8]. Limitations of the split-sample approach include creation of imprecise models [17–19], underestimated performance of the full model [17], non-efficient use of all data [17, 20], adverse effects on calibration [4, 21], and that validation only occurs on a sample of the full-dataset [20]. As a result of the limitations with the spilt-sample approach for internal validation of prognostic models, bootstrapping has been recommended as the preferred approach for assessing the internal validation of predictive models [17, 18, 20–24]. The bootstrapping approach is able to provide optimism-corrected estimates of the fit statistics. A previous comparison of the bootstrapping approach using a split-sample to predict 30-day mortality following acute myocardial infarction identified that internal validity was best estimated with bootstrapping as it provided the most stable estimates with low bias [17]*.* This research aims to determine if the predictive ability of survival and ICU admission using ICISS can be improved depending on the method used to derive ICISS and incremental inclusion of covariates.

## Method

### Linked hospitalisation and mortality data

Hospitalisation data included information on all inpatient admissions for all public and private hospitals in New South Wales (NSW), Australia during the period 1 January 2010 to 30 June 2014. Diagnoses and external cause codes were classified using the International Classification of Diseases, 10th Revision, Australian Modification (ICD-10-AM) [25]. Injury-related admissions were identified using a principal diagnosis of injury (ICD-10-AM: S00-T89). Mortality data from 1 January 2010 to 31 March 2015 from the NSW Registry of Births, Deaths and Marriages was probabilistically linked to the hospitalisation records by the Centre for Health Record Linkage.

The state of NSW, Australia covers an area of 800,628 km^2^ [26], with a population of 7.7 million [27]. NSW has had a trauma management system since 1991. This system facilitates transfer to and optimal treatment of an injured individual at the most appropriate hospital [28]. A major, level 1 trauma service is able to provide the full spectrum of care for severely injured patients from resuscitation to discharge and a regional trauma service is capable of providing care to individuals with minor to moderate injuries [29]. Regional trauma services often provide initial assessment and stabilisation of a seriously injured patient, before transfer to a major trauma service. Within NSW there are ten major trauma centres (including three paediatric) and ten regional trauma centres [30].

All hospital episodes of care related to the one injury event were linked to form a period of care (i.e. all episodes of care related to the injury until discharge from the health system). Ninety-day mortality was calculated from the date of admission of the index injury hospital admission. Where an individual was treated at more than one hospital for their injury, trauma care was considered to be delivered at the hospital where the majority of patient care was provided as defined by length of stay (LOS).

### Comorbidity identification

The Charlson Comorbidity Index [31] was used to identify 17 comorbidities using up to 50 diagnosis classifications in the hospitalisation data and a 12-month look-back period to 1 January 2009. Each comorbid condition was assigned a weight between 1 and 6 based on the risk of mortality and/or resource use and the sum of weights was used to generate a comorbidity score. A higher score is indicative of a higher likelihood of mortality and/or resource use. In addition, specific health conditions that are associated with injury risk and poor recovery [32, 33] including mental health conditions (ICD-10-AM: F20-F50), alcohol misuse and dependence (ICD-10-AM: F10, Y90, Y91, Z50.2, Z71.4, Z72.1), and drug-related dependence (ICD-10-AM: F11-F16, F19, Z50.3, Z71.5, Z72.2) were also identified using diagnosis classifications.

### Calculation of the international classification of injury severity score

For all of the index injury hospital admissions, a SRR was calculated for each injury diagnosis. A SRR represents the ratio of the number of individuals with each injury diagnosis who did not die to the total number of individuals with the injury diagnosis. The mean number of diagnoses recorded per injured individual was 1.74 (SD = 1.46; range 1–43). For each injury admission, two ICISS values were calculated: (i) multiplicative-injury ICISS where ICISS is the product of all SRRs for each of the individual’s injuries; and (ii) single worst-injury, where ICISS only includes the worst-injury (i.e. the injury diagnosis with the lowest SRR) as the single worst-injury has been shown to have good discriminatory ability for survival [10].

### Data analysis

All analyses were performed using SAS version 9.4 [34]. Logistic regression was used to examine both 90-day mortality and ICU admission as outcomes with varying predictor variables: ICISS (i.e. multiplicative or worst-injury), age group (i.e. 0–16, 17–24, 25–44, 45–64, 65–79, ≥80 years), sex, Charlson Comorbidity Index group (i.e. 0, 1–2, 3–4, ≥5 on the comorbidity index score), mental health conditions (i.e. Y/N), alcohol misuse and dependence (i.e. Y/N), drug related dependence (i.e. Y/N), trauma service level (i.e. major trauma, regional trauma, other hospital), injury mechanism, and nature of injury.

The models were assessed in terms of their ability to discriminate survivors and non-survivors using the ROC curve and the concordance statistic (c-statistic), with better discrimination scores having more area under the ROC curve. A ROC of ≥0.7 and < 0.8 as a general indication was considered to provide acceptable discrimination, a ROC of ≥0.8 and < 0.9 was considered to indicate excellent discrimination, and a ROC ≥0.9 was considered to indicate outstanding discrimination [35]. Model fit was examined using the Akaike information criterion (AIC) that indicates how close a statistical model approaches the true distribution, with lower values indicating a better fit. Goodness-of-fit was examined using the Hosmer-Lemeshow (H-L) statistic that compared predicted mortality with actual mortality, with lower values indicating better calibration. Nagelkerke’s R^2^ was used as a pseudo R-squared to provide additional information regarding goodness-of-fit of the models by indicating the proportion of outcome variance explained, ranging from 0 to 1, with values closer to one indicating higher variance explained by the model.

The full hospital-mortality data extract was used to calculate the SRRs in order to include the widest possible range of injury diagnosis codes. Therefore, to account for possible bias caused by using a single data extract for both development and testing, non-parametric bootstrapping with 200 replications was used to correct for optimism for calculating the fit statistics and 95% confidence intervals [21]. Bootstrapping involved fitting the full model and then deriving the parameter estimates which were applied to the full dataset to obtain the apparent fit statistics. A bootstrap data sample was then generated to derive fit statistics on the sample using the fitted value from the bootstrapped dataset. The fitted value was applied to the original full dataset to derive another set of fit statistics, which were used to calculate the optimism estimates. This process was replicated 200 times and then the average of the optimism estimates were subtracted from the apparent fit statistics [21].

## Results

During the study timeframe, there were 735,961 index injury admissions and 13,744 (1.9%) deaths within 90-days of hospital admission. Over half (55.6%) the injury hospitalisations were of males, with 68.0% aged < 65 years. For over three-quarters (79.7%) of hospitalisations there were no Charlson comorbidities identified. However, for individuals ≥65 years, 106,805 (45.3%) had at least one Charlson comorbidity identified. Just over one-quarter (27.8%) of hospital treatment was provided at a level 1 trauma centre. Falls and land transport crashes accounted for 45.7% of all injury mechanisms and fractures (34.1%) were the most common principal nature of injury (Table 1).

**Table 1:** Demographic characteristics of individuals with an injury-related hospitalisation, linked hospitalisation and mortality data, NSW, 1 January 2010 to 30 June 2014

	Number	Percent
Gender^a^		
Male	409,148	55.6
Female	326,804	44.4
Age group^b^		
0–16	107,833	14.7
17–24	80,908	11.0
25–44	158,514	21.5
45–64	152,913	20.8
65–79	111,351	15.1
≥ 80	124,438	16.9
Charlson Comorbidity Index group		
0	586,878	79.7
1–2	96,062	13.1
3–4	29,312	4.0
≥ 5	23,709	3.2
Mental health diagnoses^c^	69,923	9.5
Alcohol misuse and dependence	53,280	7.2
Drug-related dependence	26,479	3.6
Trauma service level		
Level 1 trauma centre	204,530	27.8
Regional trauma centre	131,987	17.9
Other hospital	399,444	54.3
Injury mechanism		
Land transport incidents	73,977	10.1
Falls	262,196	35.6
Inanimate mechanical forces	88,323	12.0
Drowning and submersion and other threats to breathing	1558	0.2
Smoke, fire and flames, heat and hot substances	7497	1.0
Poisoning	11,563	1.6
Intentional self-harm	35,204	4.8
Assault	22,600	3.1
Other and unspecified injury mechanism	233,043	31.7
Principal nature of injury		
Superficial injuries	36,518	5.0
Open wound	89,321	12.1
Fracture	250,697	34.1
Dislocations, sprains & strains	34,367	4.7
Injury to nerves and spinal cord	7334	1.0
Injury to blood vessels	3057	0.4
Injury to muscle, fascia and tendon	28,424	3.9
Injury to internal organs	28,744	3.9
Foreign body entering through natural orifice	10,019	1.4
Burns	9274	1.3
Poisoning by drugs, medicaments and biological substances	40,425	5.5
Toxic effects of substances chiefly nonmedicinal as to source	6868	0.9
Other and unspecified injuries	190,913	25.9
Intensive care unit admission	23,054	3.1
90-day mortality	13,744	1.9

^a^Gender was missing 9 injury hospitalisations

^b^Age was missing for 4 injury hospitalisations

^c^Includes depression, schizophrenia, bipolar and anxiety disorders

### 90-day mortality

**Fig. 1:**
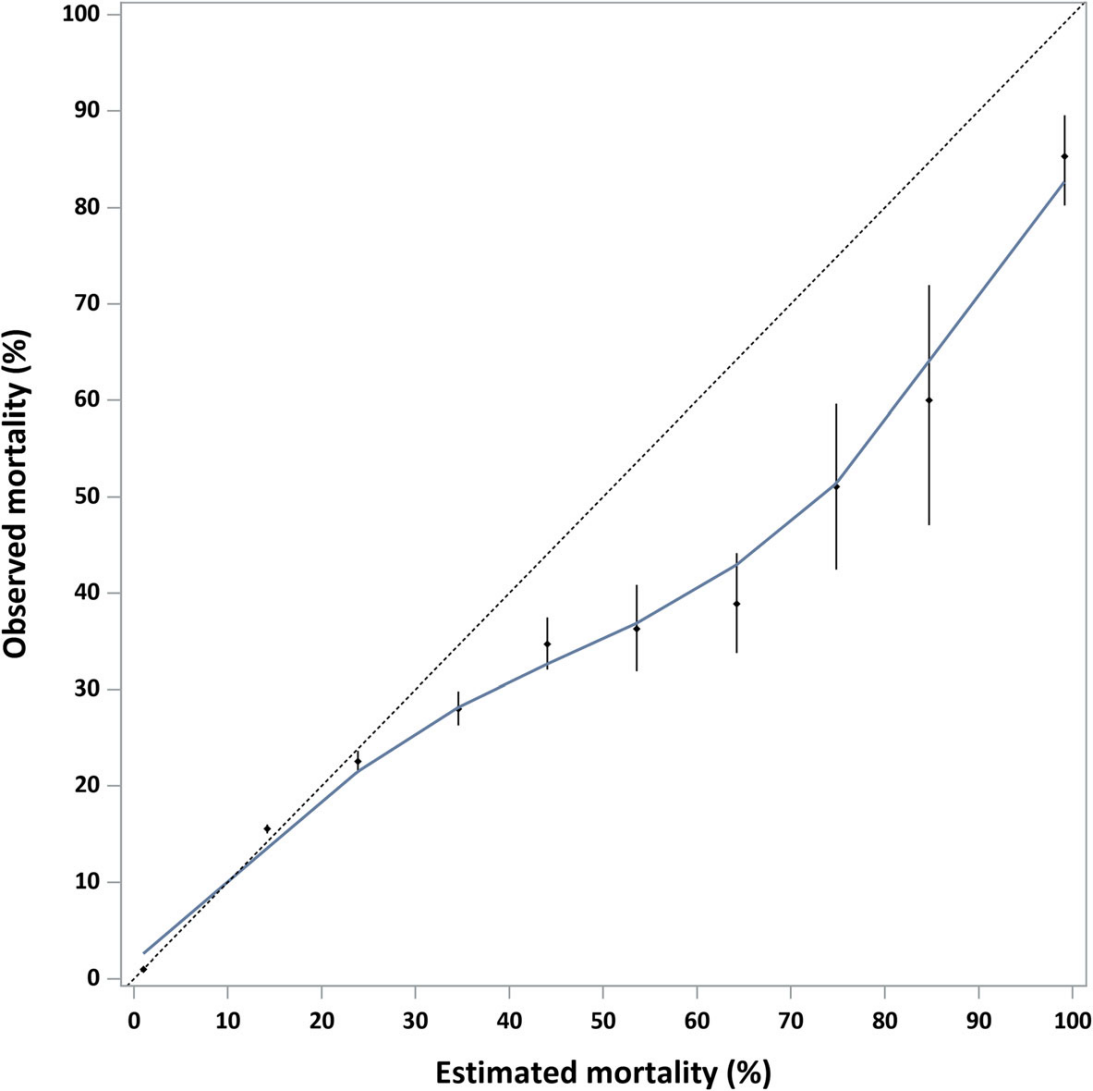
Calibration curve for best fit for single worst-injury ICISS to predict 90-day mortality, linked hospitalisation and mortality data, NSW, 1 January 2010 to 30 June 2014. Model includes: age group, gender, Charlson comorbidities, mental health conditions, alcohol misuse and dependence, drug-related dependence, injury mechanism, nature of injury and trauma centre type

The inclusion of additional predictor variables saw improvement in model assessment criteria for both multiplicative-injury and single worst-injury ICISS for 90-day mortality. As predictor variables were added, concordance improved from 0.886 to 0.917 for multiplicative-injury ICISS and from 0.894 to 0.922 for single worst-injury ICISS. The single worst-injury ICISS was identified as a better predictor of 90-day mortality than the multiplicative-injury ICISS. The best discriminatory model was generated using single-worst-injury ICISS, age group, gender, all comorbidities, trauma centre type, injury mechanism, and nature of injury that explained 33% of variation (i.e. model 8). Generally, inclusion of type of trauma centre did not improve concordance over the inclusion of comorbid conditions (Table 2). The calibration curves for 90-day mortality for all models were similar. Calibration was better for lower mortality and was very good below estimated mortality of 30% (Fig. 1).

**Table 2:** Model performance for ICISS to predict 90-day mortality, linked hospitalisation and mortality data, NSW, 1 January 2010 to 30 June 2014

	Multiplicative-injury ICISS				Single worst-injury ICISS			
90-day mortality^a^	Bootstrap-adjusted AIC (95% CI)	Bootstrap-adjusted R^2^ (95% CI)	Bootstrap-adjusted H-L statistic (95% CI)	Bootstrap-adjusted concordance (95% CI)	Bootstrap-adjusted AIC (95% CI)	Bootstrap-adjusted R^2^ (95% CI)	Bootstrap-adjusted H-L statistic (95% CI)	Bootstrap-adjusted concordance (95% CI)
Model 1: Age group, gender	104,572 (103,114, 105,888)	0.252 (0.247, 0.257)	196 (153, 235)	0.886 (0.884, 0.889)	102,256 (100,846, 103,599)	0.270 (0.265, 0.274)	134 (100, 170)	0.894 (0.892, 0.897)
Model 2: Age group, gender, CCI group	98,219 (96,972, 99,362)	0.301 (0.296, 0.306)	346 (291, 402)	0.911 (0.909, 0.914)	96,245 (95,034, 97,475)	0.316 (0.311, 0.321)	325 (267, 371)	0.917 (0.915, 0.919)
Model 3: Age group, gender, CCI group, mental health, drug, alcohol	98,187 (96,932, 99,334)	0.301 (0.296, 0.306)	392 (332, 451)	0.912 (0.910, 0.914)	96,202 (94,981, 97,447)	0.316 (0.311, 0.321)	347 (287, 393)	0.918 (0.916, 0.920)
Model 4: Age group, gender, CCI group, mental health, drug, alcohol, trauma centre	97,792 (96,515, 98,917)	0.304 (0.299, 0.309)	235 (186, 280)	0.912 (0.910, 0.914)	95,847 (94,601, 97,061)	0.319 (0.314, 0.324)	272 (223, 319)	0.918 (0.916, 0.920)
Model 5: Age group, gender, CCI group, mechanism, nature	96,731 (95,506, 97,865)	0.312 (0.308, 0.317)	315 (244, 370)	0.917 (0.915, 0.919)	95,263 (94,046, 96,449)	0.324 (0.319, 0.328)	304 (248, 356)	0.921 (0.920, 0.923)
Model 6: Age group, gender, CCI group, mechanism, trauma centre	96,513 (95,269, 97,631)	0.314 (0.309, 0.319)	281 (222, 337)	0.917 (0.915, 0.919)	94,941 (93,717, 96,108)	0.326 (0.321, 0.331)	266 (213, 309)	0.921 (0.920, 0.923)
Model 7: Age group, gender, CCI group, mechanism, nature, trauma centre	96,335 (95,099, 97,481)	0.315 (0.311, 0.320)	273 (214, 323)	0.917 (0.916, 0.919)	94,899 (93,679, 96,040)	0.326 (0.322, 0.331)	268 (222, 314)	0.922 (0.920, 0.923)
Model 8: Age group, gender, CCI group, mental health, drug, alcohol, mechanism, nature, trauma centre	96,335 (95,092, 97,472)	0.316 (0.311, 0.320)	275 (221, 323)	0.917 (0.916, 0.919)	94,904 (93,684, 96,044)	0.326 (0.322, 0.331)	273 (229, 317)	0.922 (0.920, 0.923)

^a^*CCI group* Charlson Comorbidity Index, *mental health* mental health conditions, *alcohol* alcohol misuse and dependence, *drug* drug-related dependence, *mechanism* injury mechanism, *nature* nature of injury

### ICU admission

There were 23,054 (3.1%) ICU admissions for the injured patients. Of those who were admitted to ICU, 58.8% were male, 46.7% were aged 25–64 years, 37.7% had one or more Charlson comorbidities, 27.0% were injured following a fall, 17.2% following self-harm, and 15.9% after a land transport incident. Fractures (24.1%), poisoning (19.5%), and injury to internal organs (18.3%) were the most common nature of the principal injury.

**Fig. 2:**
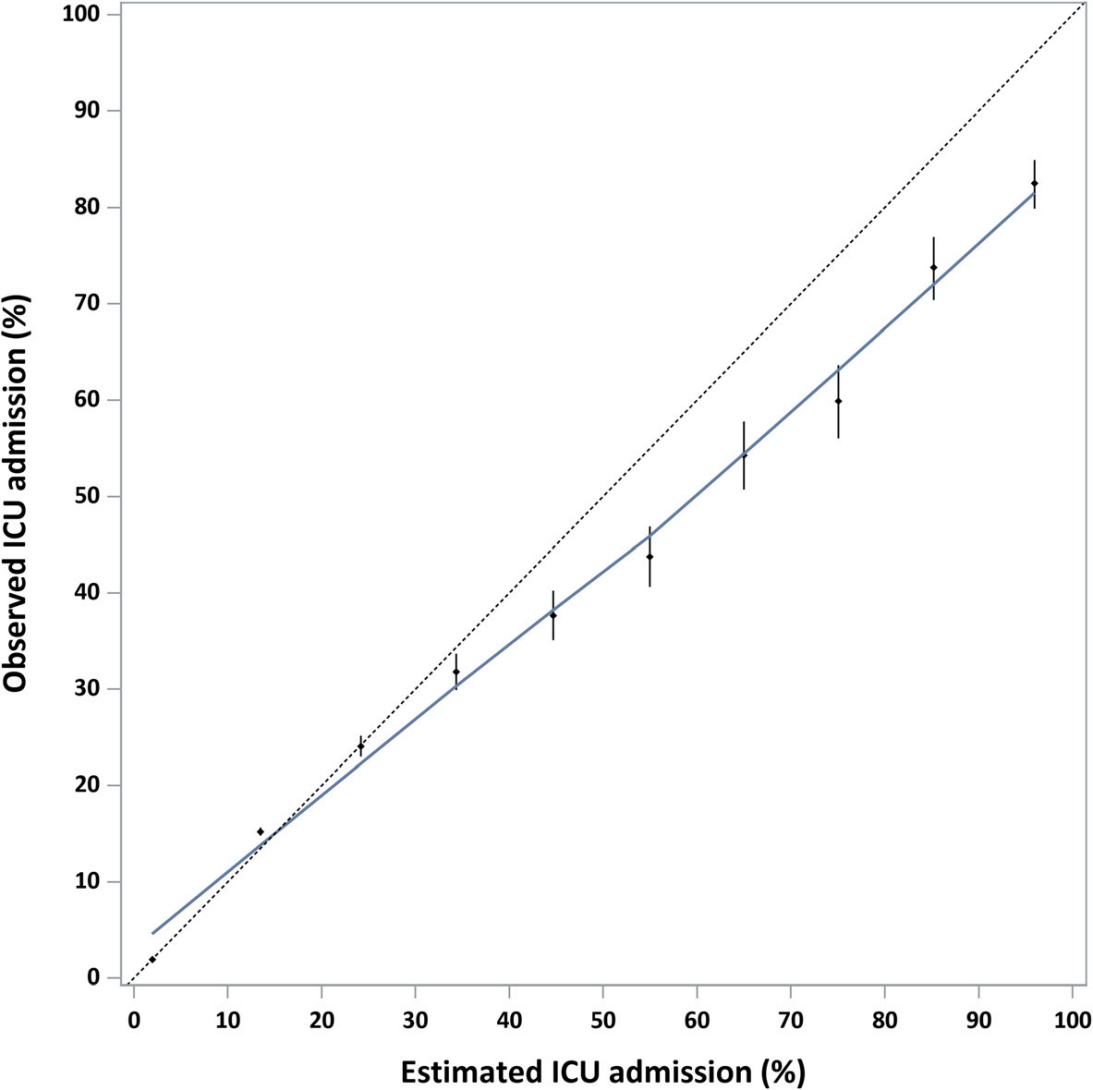
Calibration curve for best fit for multiplicative-injury ICISS to predict ICU admission, linked hospitalisation and mortality data, NSW, 1 January 2010 to 30 June 2014. Model includes: age group, gender, Charlson comorbidities, mental health conditions, alcohol misuse and dependence, drug-related dependence, injury mechanism, and nature of injury

The inclusion of comorbidities, injury mechanism and nature of injury saw improvement in model assessment criteria for multiplicative-injury and single worst-injury ICISS for ICU admission, with concordance improving from 0.763 to 0.856 for multiplicative-injury ICISS and from 0.745 to 0.841 for single worst-injury ICISS. The multiplicative-injury ICISS was identified as a better predictor of ICU admission compared to the single worst-injury ICISS. The best discriminatory model was generated using multiplicative-injury ICISS, age group, gender, all comorbidities, injury mechanism, and nature of injury which explained 25% of variation (model 7, Table 3). Calibration was better for lower ICU admission and was very good below estimated ICU admission of 40% (Fig. 2).

**Table 3:** Model performance for ICISS to predict ICU admission, linked hospitalisation and mortality data, NSW, 1 January 2010 to 30 June 2014

	Multiplicative-injury ICISS				Single worst-injury ICISS			
ICU admission^1^	Bootstrap-adjusted AIC (95% CI)	Bootstrap-adjusted R^2^ (95% CI)	Bootstrap-adjusted H-L statistic (95% CI)	Bootstrap-adjusted concordance (95% CI)	Bootstrap-adjusted AIC (95% CI)	Bootstrap-adjusted R^2^ (95% CI)	Bootstrap-adjusted H-L statistic (95% CI)	Bootstrap-adjusted concordance (95% CI)
Model 1: Age group, gender	175,710 (173,800, 177,367)	0.161 (0.156, 0.164)	1267 (1134, 1391)	0.763 (0.759, 0.766)	183,437 (181,589, 185,142)	0.119 (0.115, 0.123)	1007 (892, 1121)	0.745 (0.741, 0.748)
Model 2: Age group, gender, CCI group	173,093 (171,178, 174,762)	0.175 (0.170, 0.179)	560 (468, 652)	0.777 (0.773, 0.780)	181,083 (179,267, 182,822)	0.132 (0.127, 0.135)	520 (427, 605)	0.759 (0.756, 0.763)
Model 3: Age group, gender, CCI group, mental health, drug, alcohol	169,268 (167,500, 170,857)	0.195 (0.190, 0.199)	1083 (959, 1210)	0.809 (0.806, 0.812)	177,355 (175,605, 178,961)	0.152 (0.147, 0.156)	1333 (1170, 1495)	0.793 (0.790, 0.796)
Model 4: Age group, gender, CCI group, mechanism	162,265 (160,406, 163,818)	0.232 (0.228, 0.237)	809 (707, 897)	0.841 (0.839, 0.844)	171,083 (169,292, 172,649)	0.185 (0.182, 0.190)	619 (541, 699)	0.824 (0.822, 0.826)
Model 5: Age group, gender, CCI group, nature	160,529 (158,753, 162,033)	0.241 (0.237, 0.246)	589 (512, 661)	0.849 (0.847, 0.852)	168,908 (167,072, 170,450)	0.197 (0.193, 0.201)	617 (538, 698)	0.833 (0.830, 0.835)
Model 6: Age group, gender, CCI group, mechanism, nature	158,863 (157,039, 160,357)	0.250 (0.246, 0.255)	555 (468, 632)	0.855 (0.852, 0.857)	166,481 (164,735, 168,032)	0.210 (0.206, 0.214)	366 (301, 436)	0.839 (0.837, 0.842)
Model 7: Age group, gender, CCI group, mental health, drug, alcohol, mechanism, nature	158,474 (156,669, 159,970)	0.252 (0.248, 0.257)	572 (489, 650)	0.856 (0.854, 0.859)	165,988 (164,216, 167,490)	0.213 (0.209, 0.217)	421 (356, 492)	0.841 (0.839, 0.844)

^a^*CCI group* Charlson Comorbidity Index, *mental health* mental health conditions, *alcohol* alcohol misuse and dependence, *drug* drug-related dependence, *mechanism* injury mechanism, *nature* nature of injury

## Discussion

The ability to provide an indication of injury severity for injury hospital admissions is useful to enable surveillance of injury severity for different injury mechanisms, to clinically evaluate injured patient health outcomes, to provide an indication of injury burden in the population, and to inform clinical resource use [7, 8]. This study demonstrated that the single-worst-injury ICISS was a better predictor of 90-day mortality compared to multiplicative-injury ICISS, with the best model incorporating age group, gender, all comorbidities, trauma centre type, injury mechanism, and nature of injury. While the single-worst-injury ICISS provided the best model of 90-day mortality risk, clinical significance of this model compared to the multiplicative ICISS is not expected. However, this does confirm other research which found that an individual’s worst-injury is most influential in predicting mortality risk [10], rather than a combination of multiple injuries.

Discrimination for 90-day mortality was indicated to range from excellent to outstanding, with concordance ranging from 0.886 to 0.922 for all models. These estimates are higher than previous studies that examined the calculation of ICISS using in-hospital mortality for Australian and New Zealand injury hospitalisations using ICD-10-AM [7] and for the United States (US) using National Trauma Data Bank records and ICD-9-CM [10]. Differences in concordance scores are likely between the current study and previous Australian/New Zealand and US studies as only in-hospital mortality was considered in the previous work compared to 90-day mortality in the current study, with survival post-discharge likely to provide a better indicator of overall survival. Also, differences in the classification structure between ICD-10-AM and ICD-9-CM will account for some differences in concordance scores between the Australian and US research.

The inclusion of information on comorbidities improved concordance from excellent to outstanding for both the single worst-injury and the multiplicative-injury ICISS. Pre-existing health conditions have previously been demonstrated to increase mortality following injury, even for minor-moderate severity injuries [36]. As all hospitals in one Australian state were included in the current study, the impact of including trauma centre type as a covariate in the assessment of predictors of 90-day mortality was able to be examined. However, the inclusion of type of trauma centre did not improve concordance, model fit or variation for 90-day mortality. This seems counterintuitive and could be due to selection bias, with severely injured individuals more likely to be admitted and/or transferred to level 1 trauma centres for treatment and severely injured individuals less likely to survive their injuries irrespective of where they are treated. While an individual who is transferred to a level 1 trauma centre may provide an indicator of injury severity, the severely injured individual would need to be alive when they reached the higher level of care, otherwise there is potential to introduce immortal time bias [37].

Calibration results for 90-day mortality were better at lower levels of mortality (i.e. ≤30%). This is likely to be due to the majority of hospitalisations having low mortality (i.e. ICISS values near 1). The ICISS generated in this study indicating probability of death was overestimated for higher levels of mortality (i.e. ≥60%). Other studies have also found calibration to be better at lower levels of mortality [7, 8].

In the current study, the best model to predict ICU admission was generated using multiplicative-injury ICISS, age group, gender, all comorbidities, injury mechanism, and nature of injury. The multiplicative-injury method to calculate ICISS assumes that each injury independently affects the outcome, which may not necessarily be so when an individual sustains multiple injuries [7]. However, it is possible that the superiority of the multiplicative-injury method to predict ICU admission could be due to individuals requiring ICU admission being more likely to sustain multiple injuries. Gagne and colleagues [38] have also found that the multiplicative-injury ICISS had the best discriminatory ability for ICU admission for individuals hospitalised with traumatic brain injury. Further improvements in the predictive ability of ICU admission are likely to be gained with the addition of physiologic covariates. In particular, the inclusion of covariates was able to improve the model fit for the prediction of ICU admission.

Compared to previous Abbreviated Injury Scale (AIS)-based estimates of injury severity, injury severity scoring using the ICISS is easier and more accessible [10], able to be generated using routinely collected hospital administrative data, but is reliant on accurate classification of the worst-injury. Other indices of severity, such as the Injury Severity Score (ISS), are more resource intensive, and are used by major trauma centres in NSW. The ISS is based on an assessment of all injuries sustained and is generated using AIS scores. It is calculated as the sum of squares of the single highest AIS score in each of the three most severely injured body regions out of nine body regions [39]. Calculation of ISS requires specialist training of data coders and specialist resources. Moving to the routine clinical use of ICISS to estimate injury severity would seem to be a preferential option as comparisons of ICISS and ISS have indicated that ICISS performs as well or better than ISS [1, 5, 40].

There are several limitations of the current study. No information was available on pre-hospital treatment and care and/or use of emergency transport services that may have aided survival. There was also no information available on physiologic responses, such as Glasgow Coma Score, respiratory rate or systolic blood pressure. There were modest numbers for some injury diagnosis classifications and there is a low proportion of ICU admissions and deaths in the current study, so SRRs were based on small counts. The low proportion of deaths would have influenced the SRRs, as a higher proportion of diagnoses classifications would have survived. Likewise, for ICU admissions, with injured individuals being less likely to be admitted to ICU. However, balancing these limitations, these injuries represented all the hospital admissions and 90-day mortality in the state.

Additionally, no validation of the diagnosis classifications in the hospital administrative records was able to be conducted. This study did not use a split-sample approach on hospital-mortality data extract to develop the SRRs, instead the SRRs were developed on the original dataset to maximise the injury diagnosis classifications available to develop the SRRs, and bootstrapping was applied. The authors did investigate and perform a midpoint time-based split-sample approach for modelling and testing, with little difference in R^2^ or concordance values for ICISS to predict 90-day mortality (Additional file 1: Table S1), or to predict ICU admission (Additional file 2: Table S2). Only health conditions relevant to the hospitalisation are recorded, so it is possible that the number of health conditions experienced are under-enumerated, even with the 1-year look-back period. A longer lookback period may have been able to provide a better indication of long-term comorbid health conditions [41]. Deaths that occurred prior to hospital admission were not considered. External validation of the SRRs generated in the current study is recommended to ascertain if differences in model performance for 90-day mortality and ICU admission are replicated using the same predictor variables in other jurisdictions. Lastly, ICISS is an indicator of threat-to-life, so does not consider threat to on-going disability following injury.

## Conclusion

This study has demonstrated that the worst-injury ICISS was a better predictor of 90-day mortality and that multiplicative-injury ICISS was a better predictor of ICU admission. It also demonstrated better calibration and explained variance for both outcomes with inclusion of covariates, particularly comorbid conditions, injury mechanism and nature of injury. Moves to routinely use ICD-based injury severity measures, such as ICISS, should be considered for hospitalisation data.

## Additional files

Additional file 1: **Table S1.** Model performance for ICISS to predict 90-day mortality using split-sample approach, linked hospitalisation and mortality data, NSW, 1 January 2010 to 30 June 2014 (DOCX 16 kb)

Additional file 2: **Table S2.** Model performance for ICISS to predict ICU admission using spilt-sample approach, linked hospitalisation and mortality data, NSW, 1 January 2010 to 30 June 2014 (DOCX 16 kb)

## Abbreviations

AIC: Akaike information criterion
AIS: Abbreviated Injury Scale
H-L: Hosmer-Lemeshow
ICD: International Classification of Diseases
ICISS: International Classification of Injury Severity Score
ICU: Intensive Care Unit
LOS: Length of Stay
NSW: New South Wales
ROC: Receiver Operator Characteristic
SRR: Survival Risk Ratio
