# Supplementary material for: Identification and internal validation of models for predicting survival and ICU admission following a traumatic injury
Source: Scand J Trauma Resusc Emerg Med. 2018 Nov 12;26:95. doi: 10.1186/s13049-018-0563-5 (PMC6233597; doi:10.1186/s13049-018-0563-5)
Supplement: Supplementary file 1 — Table S1. Model performance for ICISS to predict 90-day mortality using split-sample approach, linked hospitalisation and mortality data, NSW, 1 January 2010 to 30 June 2014 (DOCX 16 kb) [file 13049_2018_563_MOESM1_ESM.docx]

**Table S1: Model performance for ICISS to predict 90-day mortality using split-sample approach, linked hospitalisation and mortality data, NSW, 1 January 2010 to 30 June 2014**

|  | **Multiplicative-injury ICISS** | | | | **Single worst-injury ICISS** | | | |
| --- | --- | --- | --- | --- | --- | --- | --- | --- |
| **90-day mortality**^1^ | **AIC** | **R^2^** | **H-L statistic** | **Concordance** | **AIC** | **R^2^** | **H-L statistic** | **Concordance** |
| Model 1: Age group, gender | 54,420 | 0.249 | 88 | 0.886 | 53,685 | 0.260 | 68 | 0.892 |
| Model 2: Age group, gender, CCI group | 51,560 | 0.291 | 219 | 0.909 | 50,932 | 0.301 | 184 | 0.914 |
| Model 3: Age group, gender, CCI group, mental health, drug, alcohol | 51,549 | 0.292 | 241 | 0.910 | 50,912 | 0.301 | 201 | 0.914 |
| Model 4: Age group, gender, CCI group, mental health, drug, alcohol, trauma centre | 51,411 | 0.294 | 210 | 0.909 | 50,776 | 0.303 | 193 | 0.914 |
| Model 5: Age group, gender, CCI group, mechanism, nature | 50,877 | 0.302 | 213 | 0.914 | 50,473 | 0.308 | 185 | 0.917 |
| Model 6: Age group, gender, CCI group, mechanism, trauma centre | 50,823 | 0.302 | 216 | 0.914 | 50,361 | 0.309 | 192 | 0.917 |
| Model 7: Age group, gender, CCI group, mechanism, nature, trauma centre | 50,742 | 0.304 | 219 | 0.914 | 50,341 | 0.310 | 189 | 0.917 |
| Model 8: Age group, gender, CCI group, mental health, drug, alcohol, mechanism, nature, trauma centre | 50,757 | 0.304 | 216 | 0.914 | 50,357 | 0.310 | 185 | 0.917 |

^1^ CCI group=Charlson Comorbidity Index; mental health=mental health conditions; alcohol=alcohol misuse and dependence; drug=drug-related dependence; mechanism=injury mechanism; nature=nature of injury.
