# Supplementary material for: Identification and internal validation of models for predicting survival and ICU admission following a traumatic injury
Source: Scand J Trauma Resusc Emerg Med. 2018 Nov 12;26:95. doi: 10.1186/s13049-018-0563-5 (PMC6233597; doi:10.1186/s13049-018-0563-5)
Supplement: Supplementary file 2 — Table S2. Model performance for ICISS to predict ICU admission using spilt-sample approach, linked hospitalisation and mortality data, NSW, 1 January 2010 to 30 June 2014 (DOCX 16 kb) [file 13049_2018_563_MOESM2_ESM.docx]

**Table S2: Model performance for ICISS to predict ICU admission using spilt-sample approach, linked hospitalisation and mortality data, NSW, 1 January 2010 to 30 June 2014**

|  | **Multiplicative-injury ICISS** | | | | **Single worst-injury ICISS** | | | |
| --- | --- | --- | --- | --- | --- | --- | --- | --- |
| **ICU admission**^1^ | **AIC** | **R^2^** | **H-L statistic** | **Concordance** | **AIC** | **R^2^** | **H-L statistic** | **Concordance** |
| Model 1: Age group, gender | 92,692 | 0.162 | 772 | 0.769 | 96,762 | 0.120 | 641 | 0.751 |
| Model 2: Age group, gender, CCI group | 91,279 | 0.176 | 257 | 0.782 | 95,474 | 0.133 | 350 | 0.765 |
| Model 3: Age group, gender, CCI group, mental health, drug, alcohol | 89,496 | 0.194 | 599 | 0.812 | 93,741 | 0.151 | 709 | 0.797 |
| Model 4: Age group, gender, CCI group, mechanism | 85,830 | 0.231 | 449 | 0.843 | 90,476 | 0.184 | 338 | 0.826 |
| Model 5: Age group, gender, CCI group, nature | 84,895 | 0.241 | 362 | 0.851 | 89,258 | 0.197 | 378 | 0.834 |
| Model 6: Age group, gender, CCI group, mechanism, nature | 84,085 | 0.249 | 324 | 0.855 | 88,059 | 0.209 | 202 | 0.840 |
| Model 7: Age group, gender, CCI group, mental health, drug, alcohol, mechanism, nature | 83,924 | 0.251 | 340 | 0.857 | 87,853 | 0.211 | 228 | 0.842 |

^1^ CCI group=Charlson Comorbidity Index; mental health=mental health conditions; alcohol=alcohol misuse and dependence; drug=drug-related dependence; mechanism=injury mechanism; nature=nature of injury.
